# Detecting heat stress: Examination of temperature-humidity index thresholds for respiration rate and body temperature in barn- and pasture-housed peripubertal dairy heifers

**DOI:** 10.3168/jdsc.2025-0790

**Published:** 2025-08-28

**Authors:** K.M. Daniels, M.D. Ellett, C.L.M. Parsons, B.A. Corl

**Affiliations:** 1 School of Animal Sciences, Virginia Tech, Blacksburg, VA 24061; 2 Department of Biology, James Madison University, Harrisonburg, VA 22807

## Abstract

If animals cannot dissipate accumulated heat from metabolism and the environment, heat stress (HS) occurs. Temperature-humidity index (THI) thresholds for peripubertal dairy heifers are not well defined, despite their known susceptibility to HS. Establishing THI thresholds in heifers housed in heat-abated freestall barns and on pasture will provide practical management metrics and inform future HS research. This study included 2 experiments in a humid continental climate: one under natural seasonal conditions and another in climate-controlled chambers. The objectives were to (1) establish THI thresholds for HS in peripubertal dairy heifers under different housing types, (2) develop prediction equations for respiration rate (RR) and body temperature in heifers using THI as the predictor variable, and (3) determine if a climate-controlled environment could result in similar biological responses and THI breakpoints as observed in peripubertal heifers housed in summer pasture. In the first experiment, RR, vaginal body temperature, and THI were recorded twice daily for a total of 14 d in 12 peripubertal heifers (291 ± 19 kg BW, 281 ± 6 d of age; mean ± SD). Each heifer experienced 7 d of housing in a naturally ventilated barn with fans above the stalls and 7 d of outdoor pasture housing, in a crossover fashion. Simple linear and segmented regression were used to develop prediction models for RR and body temperature, using THI as the predictor variable. Simple linear regression best modeled RR and body temperature response to THI in heifers housed in a barn with fans above the freestalls when THI ranged from 64 to 82. In contrast, a segmented regression with a THI threshold of ∼74 best reflected RR and body temperature response to THI for pasture-housed heifers when THI ranged from 62 to 88. After THI reached the threshold of 74 without remediation, signs of HS escalated abruptly. A second experiment was conducted to determine if housing in a climate-controlled chamber could result in similar biological responses and THI breakpoints as observed in heifers housed in summer pasture. For this, RR and body temperature relative to observed THI were monitored twice daily in a subset of 5 heifers from experiment 1 (340 ± 16 kg BW; mean ± SD) that were housed in a climate-controlled chamber. In this setting, THI was increased incrementally every 24 h over a 5-d period, from ∼65 to 80. Similar to pasture housing, RR and body temperature abruptly increased with each unit increase in THI beginning at THI of 74 for RR and 76 for body temperature. Collectively, these experiments confirm that peripubertal dairy heifers exhibit classic signs of HS, with RR and body temperature increasing sharply near THI 74 in both pasture and climate-controlled environments. Heifers housed in environments with heat abatement features do not respond to increased THI in the same manner; biological responses to increased THI remain linear. Regardless of housing environment, heifers weighing ∼325 kg should be monitored more closely for signs of HS as THI approaches 74 to minimize the risk of HS-related production losses.

## Highlights

• The THI threshold for increased RR and BT in pasture heifers is ~74.
• Created THI-based equations to predict RR and BT in barn and pasture housed heifers.
• The RR and BT versus THI (64–82) best fit by linear regression in cooled freestall heifers.
• Without cooling, RR and BT rose sharply at THI 74 and 76 in indoor-housed heifers.

## Graphical Abstract

**Figure:**
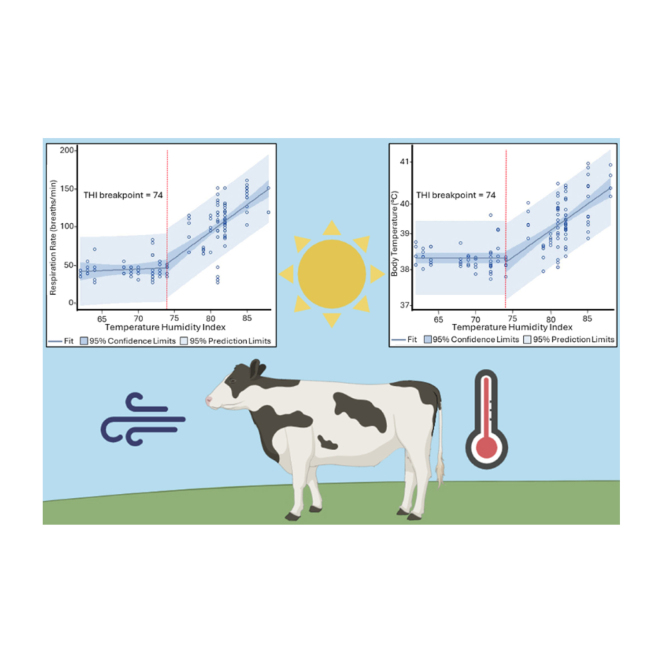
**Summary:** Heat stress (HS) occurs when an animal accrues a heat load that disrupts the regulation of internal temperature. The temperature-humidity index (THI) threshold for HS in dairy heifers is not well defined in the literature. The objectives of the current work were to establish THI thresholds for HS in dairy heifers and to develop predictive equations for respiration rate (RR) and core body temperature (BT). It was observed in a series of 2 experiments that a THI threshold of 74 elevated RR and BT in heifers housed in a heat-abated freestall barn, on pasture, and in a climate-controlled chamber. Simple linear regression best modeled RR and BT response to THI in heifers housed in a freestall barn, and a segmented regression best reflected RR and BT response to THI for pasturehoused heifers. Heifers should be monitored for signs of HS when THI approaches 74.

Heat stress imposes a significant economic burden on the US dairy industry, producing estimated annual losses exceeding $1 billion (Key and Sneeringer, 2014). If animals cannot dissipate accumulated heat from metabolism and the environment, heat stress (**HS**) occurs. Unmitigated, HS has detrimental effects on nutrient utilization. Inefficient nutrient utilization in heifers negatively affects growth, prolongs the nonproductive rearing period, and increases nutrient losses to the environment, adding to costs. Despite known susceptibility to HS, the temperature-humidity index (**THI**) threshold for dairy heifers is not well defined in the literature, as it is for lactating cows (see review by Tao et al., 2020), dry cows (Ouellet et al., 2021), and even dairy calves (Dado-Senn et al., 2020; Dado-Senn et al., 2023). Establishing THI thresholds in peripubertal heifers housed in heat-abated freestall barns and on pasture will provide practical management metrics and aid in the design and implementation of future HS research studies that use climate-controlled chambers. The objectives were to (1) establish THI thresholds for HS in peripubertal dairy heifers under different housing types in a humid continental climate, (2) develop prediction equations for respiration rate (**RR**) and body temperature in heifers using THI as the predictor variable, and (3) determine if a climate-controlled environment could result in similar biological responses and THI breakpoints as observed in heifers housed in summer pasture to allow for controlled chamber studies when experiments on pasture are infeasible.

All procedures were approved by the Virginia Tech Institutional Animal Care and Use Committee (protocol #24-079). This study was conducted through 2 experiments: the first under natural seasonal conditions, and the second in a controlled climate environment. The first experiment used a crossover design to capture environmental and animal measurements, which were then used in subsequent regression analyses to (1) establish THI breakpoint(s) at which dairy heifers become heat-stressed and (2) develop prediction equations for animal metrics (e.g., RR, body temperature) based on THI. Once THI breakpoints were established and THI prediction equations constructed, a second experiment was conducted to determine if housing in a climate-controlled chamber, without wind or solar radiation but with controlled THI, could result in similar biological responses and THI breakpoints as observed in heifers housed in summer pasture.

The first experiment was conducted from June 17 to July 15, 2024, at the Virginia Tech dairy farm located in Blacksburg, VA. Blacksburg has a humid continental climate with hot summers. The experiment contained a 7-d period 1, 14-d rest period, and a 7-d period 2. Twelve peripubertal Holstein heifers (291 ± 19 kg BW, 281 ± 6 d of age; mean ± SD) with prior experience using freestalls, consuming a TMR, and grazing on pasture were enrolled. Before project start, all heifers were housed in a single fenced pasture centered at 37°12′01ʺN 80°34′23ʺW, as per normal management practice in a paddock with 16 additional contemporaries. On June 16th, 2024, experiment heifers were randomized into 1 of 2 sequences of housing locations for periods 1 and 2, respectively (pasture-barn, or barn-pasture) by drawing heifer identification numbers from a hat. From 0800 to 0915 h on June 17, 2024, all heifers were weighed and each fitted with a vaginal temperature logging device like that described by Burdick et al. (2012), set to record body temperature at 1-min intervals. Heifers initially assigned to the pasture treatment were returned to the adjacently located original pasture, whereas those initially allocated to the barn treatment were transported by trailer a short distance (<5 min) to their designated pen within a naturally ventilated barn with fans above the freestalls that were familiar to them. All heifers were in place by 1000 h on June 17, 2024.

The pasture paddock measured ∼40,700 m^2^, had 2 automatic waterers, a concrete feedbunk, and minimal natural shade. In addition to pasture, which consisted of mixed grasses, heifers had access to a TMR that was delivered once daily in the feedbunk at ∼0945 h. The pen within the sidewall-curtained freestall barn measured ∼247 m^2^ total and contained 30 sand-bedded stalls with ∼5 m^2^ of resting space per heifer. The geographic center of the pen was 37°12′05ʺN 80°34′33ʺW. In addition to the freestalls, the pen included a waterer and a feedbunk with headlocks. Six overhead fans were programmed to automatically turn on and off at an ambient temperature of 21°C, as per normal herd management. When fans were on, air velocity within the direct path averaged 3.2 m/s at 155 cm above the stall surface (data not shown). Sidewall curtains and barn doors were always open. Fresh TMR was delivered once daily at ∼0945 h. The TMR provided at both housing locations was the same standard herd ration, with daily delivery amounts adjusted by location to support an average daily BW gain of ∼1 kg. Feed intake was not measured.

Twice daily measurements for period 1 began on June 17 at 1530 h and were completed at 1530 h on June 24, 2024. Measurement times of ∼0730 h and 1530 h were chosen to represent the approximate lowest and highest THI points for a June day in Blacksburg. While obtaining measurements, time of day was noted with accuracy to the nearest minute. From a designated spot within each housing location, a hand-held meter (WBGT Heat Stress Meter, model R6200, Reed Instruments, Wilmington, NC) was used at an approximate height of 155 cm to obtain a representative dry bulb temperature (**T_db_**) and relative humidity (**RH**) reading. The stated accuracies of the meter were ± 1°C and ± 3% RH. Later, T_db_ and RH values were used to compute corresponding THI with the formula: THI = (0.8 × T_db_) + [(RH/100) × (T_db_ − 14.4)] + 46.4. At each visit, RR per heifer was counted over 30 s and doubled to obtain breaths per minute by one of 4 trained observers. Care was taken to not disturb heifers while measurements were obtained and position (standing versus laying) was documented. Vaginal temperature loggers were removed from each heifer at the end of period 1. Time-stamped vaginal body temperature data corresponding to time of RR measurements were retrieved from each heifer's logged data after device removal.

Heifers stayed in the initial housing location until the morning of July 8, 2024. That morning, vaginal temperature logging devices were re-installed. Heifers were then moved to designated period 2 location. Period 2 measurements were obtained from July 8 to July 15, 2024 in the manner reported for period 1.

Heifers that finished experiment 1 in the freestall barn were used for experiment 2, which was conducted to determine if housing in a climate-controlled chamber, without wind or solar radiation but with controlled THI, could result in similar biological responses and THI breakpoints as observed in heifers housed in summer pasture (experiment 1). For this, peripubertal heifers (n = 6; 340 ± 16 kg BW; mean ± SD) were acclimated to the Metabolism Research Laboratory (**MRL**) at the Virginia Tech dairy (Blacksburg, VA) over a period of 2 d, beginning on July 23, 2024. Vaginal temperature loggers were re-inserted on the evening of July 24, 2024. Beginning at 0600 h on July 25, all heifers remained in a single room (i.e., chamber) within the MRL and were tethered in individual sawdust-bedded stalls. Water and TMR were always available. Beginning at 0600 h on each of 5 successive days and persisting for 24 h/d, the MRL facility was programmed to sequence through planned THI setpoints of 65, 70, 75, 80, and 85. Twice daily measurements were recorded for experiment 1. The experiment ended on July 29, 2024, after 10 observations per heifer.

With data obtained in experiment 1, we generated 4 datasets. Two datasets were made for barn-housed heifers; one contained RR and THI data and the other contained body temperature and THI data. Two datasets for pasture-housed heifers were similarly constructed. The pasture datasets each contained 131 observations, and the barn datasets each contained 133 observations. Missing data points are attributed to early removal of temperature monitoring devices in heifers; when temperature monitoring devices were removed early, corresponding RR data were also removed. All analyses were performed in SAS, version 9.4 (SAS Institute Inc., Cary, NC). Each dataset was subjected to simple linear regression analysis with PROC REG and segmented regression analysis with PROC NLIN. In each dataset and within each type of regression analysis, THI was used as the sole explanatory variable and RR and body temperature were used as sole dependent variables. The purpose of performing each type of regression analysis was to ascertain if a THI breakpoint could be established within each dataset. A THI breakpoint was said to exist when segmented regression models yielded higher R^2^, lower residual sums of squares (**RSS**), and lower root mean square errors (**RMSE**) compared with simple linear regression models, indicating a rapid increase in either RR or body temperature at a given THI threshold. If segmented models failed to converge or did not markedly improve model fit based on R^2^, RSS, and RMSE, the segmented model was rejected in favor of the simple linear model with no breakpoint.

For segmented regression analysis, the Marquardt method was used to find the best-fitting parameters for each of the 2 segmented models we developed for THI breakpoint determination. Within the NLIN procedure and for each model, we specified initial estimates for the following parameters: intercept of the first segment (b_01_), slope of the first segment (b_11_), intercept of second segment (b_02_), slope of second segment (b_12_), and THI breakpoint. We also included a binary variable, THI_indicator_, which has a value of 1 if THI ≥ THI_breakpoint_, and 0 otherwise. The model equation is as follows:

y = b_01_ + b_11_ × THI + THI_indicator_ × [b_02_ − b_01_ + b_12_ × (THI − THI_breakpoint_)].

In experiment 2, data from one heifer were excluded due to signs of skin irritation and an abnormally elevated baseline respiration rate (∼1.7 fold higher baseline RR), which were not observed in experiment 1. Remaining data from 5 heifers were used. Actual THI obtained for each of the 5 d in the MRL were (a.m./p.m.) 64.7/64.8, 65.1/67.3, 72.4/73.6, 76.6/77.6, and 78.9/80.6. These realized THI values were entered into a dataset along with corresponding RR and body temperature values by heifer. Dependent variables RR and body temperature were subjected to simple linear and segmented regression analyses as for experiment 1. Model fit, parameter estimates, 95% confidence limits, 95% prediction limits, R^2^, RSS, and RMSE for the best-fitting models in experiment 1 are reported in Figure 1 and Table 1; for experiment 2, these are reported in Figure 2 and Table 1.

**Figure 1:**
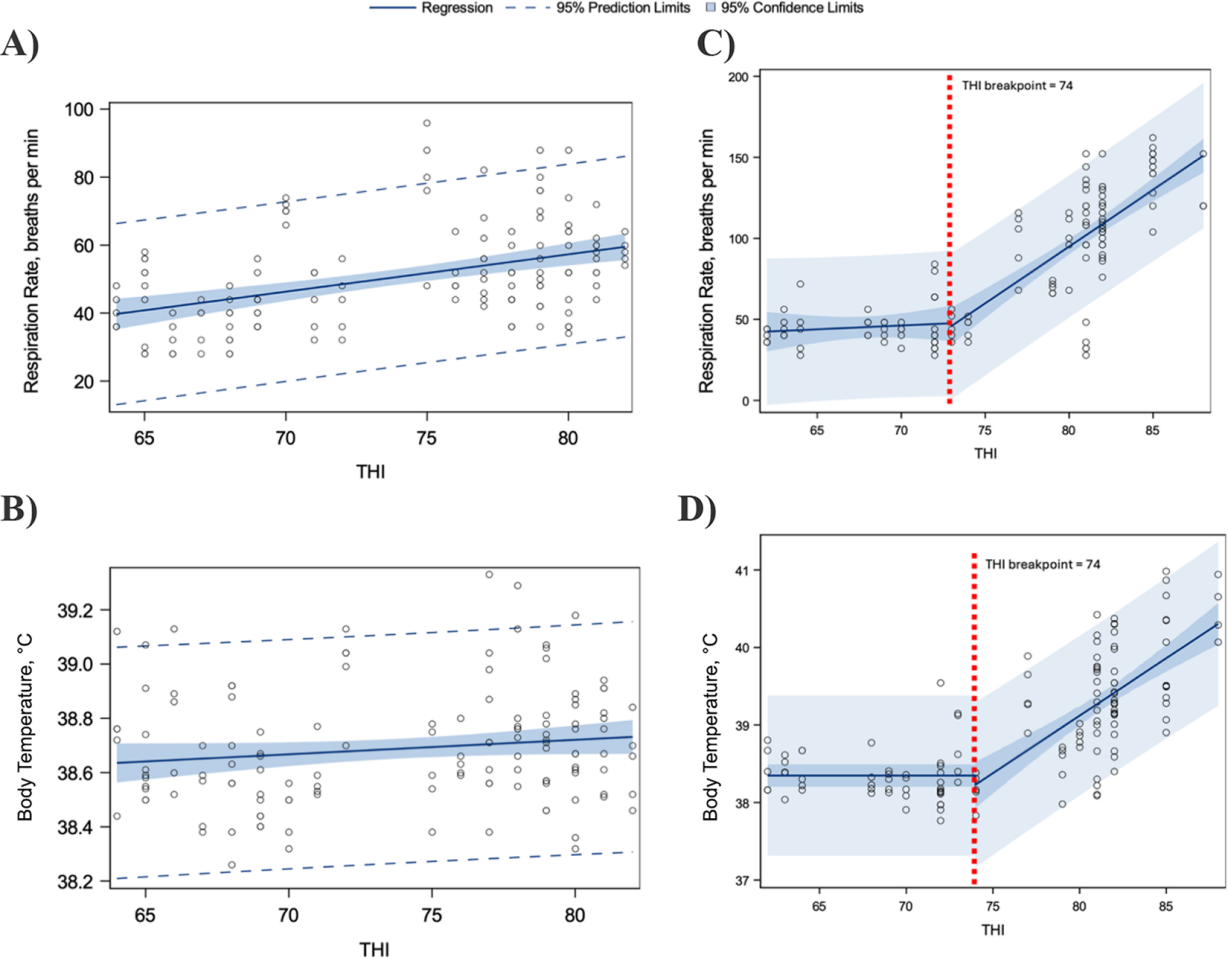
Simple linear regression plots for (A) respiration rate and (B) body temperature in Holstein heifers (n = 12; 291 ± 19 kg, 281 ± 6 d of age at start of experiment; mean ± SD) housed 7 d in a heat-abated freestall barn, observed twice daily. Segmented regression plots for (C) respiration rate and (D) body temperature in the same Holstein heifers (n = 12) housed 7 d on pasture, observed twice daily. In all plots, circles represent individual observations of x and y variables. Temperature-humidity index (THI) breakpoints were not detected for respiration rate or body temperature for heifers housed in a heat-abated freestall barn (A, B). For pasture-housed heifers (C, D), red dashed lines represent the THI breakpoint at which the response changed abruptly with increasing THI.

**Table 1:** Model-derived parameter estimates, root mean square errors (RMSE), residual sum of squares (RSS), R^2^, for simple linear and segmented regression analyses for the dependent variables respiration rate and body temperature at each of 3 locations (heat-abated freestall barn, n = 12; pasture, n = 12; or in a climate-controlled chamber with no heat abatement, n = 5)^1^

Item	Heat-abated freestall barn		Pasture		Climate-controlled (no heat abatement)^2^	
	Respiration rate, breaths/min	Body temperature, °C	Respiration rate, breaths/min	Body temperature, °C	Respiration rate, breaths/min	Body temperature, °C
Regression type	Simple^3^, ^4^	Simple^3^	Segmented^3^, ^5^	Segmented^3^	Segmented^3^	Segmented^3^
Total observations, n	133	133	131	131	50	45
Effective sample size,^6^ n	41	41	41	41	18	18
Parameter						
b_01_	−30.6624	38.2947	17.2796	38.348	−27.4755	38.5293
b_11_	1.098	0.0053	0.4084	0	1.0251	0.0001
b_02_	—	—	22.7957	38.23	−33.3698	38.4949
b_12_	—	—	6.5862	0.1483	10.7061	0.2669
THI_breakpoint_	—	—	74	74	74	76
RMSE	13.288	0.2126	21.6716	0.5084	12.6074	0.2598
RSS	23,131	1.80E-12	61,525	33.8705	7,947	3.039
R^2^	0.1925	0.0214	0.704	0.5784	0.8280	0.4723
r	0.458	0.135	0.789	0.692	0.820	0.507
*P*	0.001	0.110	0.001	0.001	0.001	0.001
A priori power^7^	0.83	0.15	0.99	0.99	0.99	0.99

1 Data from the heat-abated freestall barn and pasture are from 12 heifers measured twice daily for 7 d in each location. Data from heifers housed in a climate-controlled chamber with no heat abatement are from 5 heifers measured twice daily for 5 d. Best-fitting model solutions are shown. Simple linear models do not contain temperature-humidity index (THI) breakpoints, whereas segmented models do.

2 Measurements within a climate-controlled chamber where THI was changed every 24 h over 5 d, from approximately THI 65 to 80.

3 Model equation: y = b_01_ + b_11_ × THI + THI_indicator_ × [b_02_ − b_01_ + b_12_ × (THI − THI_breakpoint_)], where THI_indicator_ is 1 if THI ≥ THI_breakpoint_, and 0 otherwise.

4 Example calculation for an observed THI of 71. Predicted respiration rate (breaths/min) = −30.6624 + 1.098 × 71 = 47.

5 Example calculation for an observed THI of 81. Predicted respiration rate (breaths/min) = 17.2796 + 0.4084 × 81 + 1 × [22.7957 − 17.2796 + 6.5862 × (81 − 74)] = 102.

6 Effective sample size accounts for correlation between repeated observations within heifer; 12 heifers (n = 12), 11 observations per heifer to account for missing observations (m = 11), and an assumed intraclass correlation of 0.22 (ρ = 0.22). Effective sample size (n_eff_) was calculated using the formula: n_eff_ = (n × m)/[1 + (m − 1) × ρ].

7 For simple regression models, observed power was calculated using proc power within SAS (specifications: type III exact *F* test, single predictor variable, α = 0.05, effective sample size, R^2^ of full model). For segmented regression models, power was estimated via Monte Carlo simulation (1,000 iterations per model) to assess the ability to detect the threshold-dependent interaction effect (parameter b_12_, i.e., change in slope above the THI breakpoint), using model-specific parameters, RMSE, and effective sample sizes.

**Figure 2:**
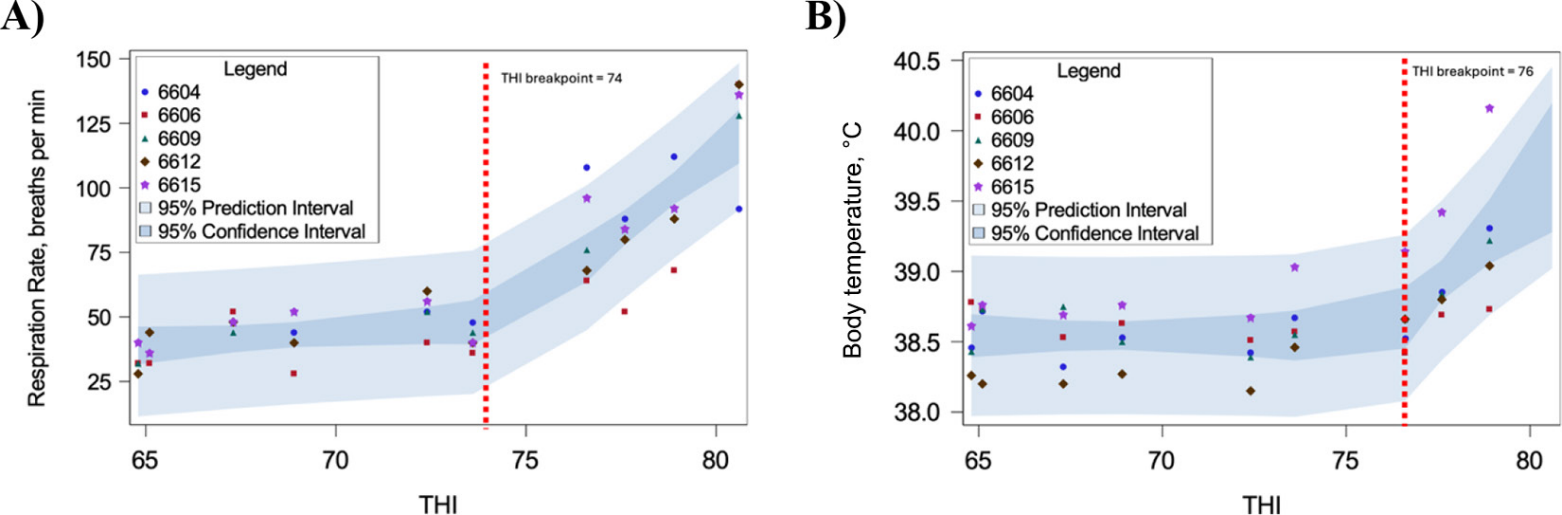
Segmented regression plots for (A) respiration rate and (B) body temperature in Holstein heifers (n = 5; individual heifer identification numbers plotted) housed in a climate-controlled chamber where temperature-humidity index (THI) was changed every 24 h over 5 d, from approximately THI 65 to 80. Respiration rate, vaginal body temperature, and THI were recorded twice daily. Red dashed lines represent the THI breakpoint at which the response changed abruptly with increasing THI.

Our first objective was to establish THI threshold(s) in a humid continental climate at which signs of HS are observed in peripubertal dairy heifers housed in a heat-abated freestall barn and on pasture. No THI thresholds for RR or body temperature were detected for freestall barn–housed heifers (Figure 1A–B; Table 1). The model for RR in heifers housed in heat-abated freestall barns was adequately powered (Table 1). In contrast, the best-fitting model for predicting body temperature in heifers housed in heat-abated freestall barns was underpowered (Table 1). Therefore, we do not provide sufficient evidence to support or refute a relationship between body temperature and THI in this group. In the heat-abated freestall barn, despite elevated THI, heifers averaged RR and body temperature 50 ± 14.2 breaths/min and 38.71 ± 0.25°C (mean ± SD). Although not included in our model, RR appeared similar between lying and standing postures based on visual inspection of raw means and SD (lying: 68 ± 35.6 breaths/min; standing: 66 ± 33.2 breaths/min) regardless of housing conditions. Lying and standing postures represented 53.4.% and 46.6% of our RR measurements, respectively. We note that posture may influence RR as animals behaviorally regulate heat load, as previously documented (Nordlund et al., 2019; Pinto et al., 2020).

In pasture-housed peripubertal heifers, we detected a THI threshold of 74 for both RR and body temperature in (Figure 1 C–D; Table 1). At a THI of 74, both RR and body temperature abruptly increased with each unit increase in THI in a humid continental climate. These changes were best represented by segmented, as opposed to simple, linear models (Figure 1). Similar regression methods have been used to determine THI breakpoints in dairy calves (Dado-Senn et al., 2020, 2023), dry cows (Ouellet et al., 2021), and lactating cows (Pinto et al., 2020). In young dairy calves (2 to 42 d old), RR begins to rise near a THI of 65 to 69, whereas rectal temperature increases at approximately THI 67 to 69 (Dado-Senn et al., 2020, 2023). Ouellet et al. (2021) reported abrupt increases in RR and rectal temperature in dry cows at a THI of 77. The commonly accepted THI threshold for reduced milk production in lactating cows is near 68 (Zimbelman et al., 2009). Pinto et al. (2020) further examined the influence of posture on THI thresholds in lactating cows, reporting values ranging from 65 (RR in lying cows) to 72 (heart rate, regardless of posture). Because THI is calculated from ambient temperature and RH, the thresholds reported by us and others appear to be applicable across different climate types, assuming that animals' physiological characteristics are appropriately considered. Thus, our THI threshold findings for pasture-housed heifers align with, and indicate, decreased thermal tolerance as dairy cattle increase in size and eventually enter lactation, where metabolic heat production associated with DMI and milk production both lower THI threshold.

Our second objective was to develop prediction equations for RR and body temperature in heifers housed in a heat-abated freestall barn and on pasture using THI as the predictor variable. The equations we derived can predict RR and body temperature if THI is known (Table 1). Predicting body temperature is practically useful because a rise of 1°C or less in rectal temperature can reduce productive performance in livestock species (Kadzere et al., 2002). Our equations could likely be improved with additional observations from a wider range of environments and heat abatement strategies with heifers of varying BW, particularly at THI >74. Also, the THI calculation we used does not account for dewpoint, windspeed, or solar radiation as incorporated in some other heat indices (Buffington et al., 1981; Mader et al., 2010; Wang et al., 2019). These added metrics could likely enhance predictive accuracy for both freestall barn and pasture-housed heifers, though they are more cumbersome to collect.

Nonetheless, our findings provide valuable, immediately applicable information to support high standards of animal welfare for dairy heifers with similar characteristics to those used here during periods of elevated ambient temperature and RH. For example, during housing audits, T_db_ and RH can easily be measured to calculate THI. When combined with housing type (e.g., ventilated barn, pasture), THI can serve as a practical tool for informing management decisions. Importantly, the likelihood of dairy heifers experiencing HS is poised to increase annually due to the rising number of extreme heat events in most US states, including top dairy states, because of climate change (EPA, 2023). We acknowledge that THI thresholds may differ for recently weaned heifers or pregnant heifers, and future research on those additional age subclasses would be beneficial.

Our third objective was to determine if housing in a climate-controlled chamber could result in similar biological responses and THI breakpoints as observed in heifers housed in summer pasture. The MRL facility did not meet the highest desired THI setpoint of 85 (Figure 2), but because calculations were based on observed THI, the impact on meeting the overall objective was minor. In our dataset, we showed that RR and body temperature abruptly increased with each unit increase in THI beginning at THI of 74 for RR and 76 for body temperature (Figure 2). These values closely match those observed in pasture-housed heifers (Figure 1C–D), indicating that the climate-controlled chamber effectively elicited a comparable HS response at similar THI thresholds. This supports the use of climate-controlled chambers for studying HS in peripubertal heifers. We note that the slightly higher THI threshold for body temperature in the chamber may be due to chronic exposure to elevated THI, which differs from the more variable conditions on pasture. A lower threshold might be detected under cyclical HS conditions. Additionally, a visual comparison of Figure 1, Figure 2 highlights the practical value of heat abatement strategies, such as fans: RR and body temperature increased abruptly in the chamber (experiment 2), but not in the mechanically and naturally ventilated freestall barn used in experiment 1.

Our collective observations across 2 experiments confirm that peripubertal dairy heifers weighing ∼325 kg exhibit classic signs of HS when housed in conditions where THI meets or exceeds ∼74 without heat abatement (Figure 1, Figure 2). Both RR and body temperature increased sharply near THI of 74 in pasture housing in a humid continental climate, and near THI of 74 when housed in a climate-controlled chamber without heat abatement. Similar physiological responses (i.e., increased RR and increased body temperature) have been noted in 135- to 180-kg bulls subjected to HS conditions (O'Brien et al., 2010, Hossein Yazdi et al., 2016).

These THI thresholds can, for now, be generalized to large-breed dairy heifers of similar weight reared in humid continental climates with hot summers under housing conditions comparable to those described in this study.
